# Individualized Algorithm-Based Intermittent Hypoxia Improves Quality of Life in Patients Suffering from Long-Term Sequelae After COVID-19 Infection

**DOI:** 10.3390/jcm14051590

**Published:** 2025-02-26

**Authors:** Josephine Schultz Kapel, Rasmus Stokholm, Brian Elmengaard, Zahra Nochi, Rikke Jentoft Olsen, Casper Bindzus Foldager

**Affiliations:** 1 SANA Medical Systems, 8240 Risskov, Denmark; 2 Research Unit for Molecular Medicine, Department of Clinical Medicine, Aarhus University and Aarhus University Hospital, 8200 Aarhus N, Denmark; 3 Danish Pain Research Center, Department of Clinical Medicine, Aarhus University, 8200 Aarhus N, Denmark

**Keywords:** COVID-19, post-COVID-19 condition, long COVID, long-term sequelae, intermittent hypoxic conditioning, pain, quality of life

## Abstract

**Background/Objectives**: Post-COVID-19 condition (PCC), also known as long COVID, has emerged as a recognized syndrome affecting millions of people worldwide, significantly impairing their quality of life. Currently, no effective therapeutic options are available to manage this condition. The objective of the present study was to evaluate the long-term effects of personalized, algorithm-based intermittent hypoxia–hyperoxia conditioning (IHHC) on quality of life and pain in patients with PCC. **Methods**: This open-label cohort study included 199 PCC patients, aged 11–87 years (female-to-male ratio: 67:33) and experiencing moderate-to-severe fatigue, between 1 January 2020 and 31 December 2023. Each patient received an algorithm-based treatment plan tailored to their demographics, symptom duration, and baseline pain (NRS) and quality of life (SF-36) scores. Patients received an average of six treatment sessions (range: 2–21), each consisting of intermittent hypoxic–hyperoxic cycles, with hypoxia (9–13% O_2_) lasting 3–8 min and hyperoxia (34–36% O_2_) lasting 1–3 min. The primary outcomes were changes in the NRS and SF-36 scores at the 6-week and 6-month follow-ups. **Results**: At the 6-week follow-up after treatment initiation, the SF-36 scores increased by 102 points (*p* < 0.001, 95% CI: 78.4–127), and this improvement persisted at the 6-month follow-up (Δ106, *p* < 0.001, 95% CI: 57.0–154). Pain was reduced by 28–32% at both follow-up time points, exceeding the clinically relevant threshold. Health transition scores indicated a patient-perceived improvement in health status. **Conclusions**: In this study, a personalized, algorithm-based IHHC alleviated pain and improved quality of life in patients suffering from persistent long-term sequelae after COVID-19 infection. The effects were sustained for up to six months. Further research is warranted to elucidate the mechanisms underlying IHHC’s therapeutic effects in this patient population.

## 1. Introduction

By June 2024, the World Health Organization (WHO) had reported more than 775 million confirmed cases of coronavirus disease 2019 (COVID-19) [1]. Although COVID-19 has been declared no longer a global health emergency [2], the long-term sequelae of the disease persist. Post-COVID-19 condition (PCC), also known as long-COVID or post-acute sequelae of COVID-19 (PACS), is a globally recognized syndrome. According to the WHO, PCC is characterized by symptoms that persist or emerge three months after an initial SARS-CoV-2 infection and continue for at least two months without an alternative explanation [3]. With over 12% of COVID-19 cases progressing to PCC [4], the condition presents a significant global health burden.

PCC has been shown to affect all organ systems [5,6,7,8], leading to an immense symptom burden for patients and strongly reducing their quality of life [9]. A research study led by the National Institutes of Health (NIH) found the most common symptoms to be post-exertional malaise (PEM) (87%), fatigue (85%), cognitive impairment including brain fog (64%) and dizziness (62%), gastrointestinal symptoms (59%), palpitations (57%), symptoms relating to hearing (46%), and joint pain (42%) [10]. Among patients who are symptomatic two months post-infection, 85% experience symptoms beyond one year [8,10]. Several pathophysiological mechanisms have been proposed to underlie the development of PCC, including sustained inflammation with or without persistent SARS-CoV-2 viral presence [11,12] or the reactivation of latent viruses [13], autoimmunity [14,15], endothelial dysregulation and micro-thrombosis leading to reduced tissue repair and ischemia [5,16,17,18], mitochondrial dysfunction [19,20,21,22], gastrointestinal microbiome dysbiosis [23,24], and dysautonomia [25,26]. A multifactorial group of mechanisms likely underlies each case of PCC, driving the multiorgan syndrome with comprehensive interindividual heterogeneity.

The ability to sense the partial pressure of oxygen is of vital importance to all aerobic cells and the organism as a whole, as hypoxia can be detrimental to the organism. Nevertheless, intermittent hypoxia conditioning (IHC), defined as controlled cyclic exposures to hypoxia (FiO_2_ = 9–13%) followed by normoxia (FiO_2_ = 21%) or hyperoxia (FiO_2_ = 30–40%), has been shown to enhance the organism’s resilience and performance [27,28,29,30]. IHC can be used as preconditioning, preparing the organism for subsequent injury, or as postconditioning, reducing damage and promoting recovery [29]. The effects of IHC have been well studied in cellular and animal disease models and are proposed to be the result of (1) mitochondrial restoration, (2) improved pro- and antioxidant balance, (3) anti-inflammatory responses, (4) stem cell migration and tissue regeneration, (5) improved vascular function, and (6) the restoration of autonomic regulation [27,28]. Given the overlap between IHC mechanisms and the pathophysiological processes associated with PCC, we hypothesize that systemic stimulation with intermittent hypoxia could serve as a potential treatment for PCC patients.

Numerous reviews [27,29,30] describe the use of IHC in disease prevention and treatment. In clinical settings, IHC has shown promising effects in diseases of the nervous system [31,32,33,34,35,36,37], the cardiovascular system [38,39,40,41,42,43,44,45,46,47,48,49,50,51], the respiratory system [52,53,54], and the musculoskeletal system [34,36,37,48,49,53]. With no severe side effects reported in these studies, IHC appears to be a safe and promising approach for the prevention and treatment of both local and systemic diseases.

Based on the principle of hormesis, hypoxic therapy is dose-dependent: low-dose controlled exposures yield stimulatory effects and restore homeostasis, whereas high-dose exposures that exceed the organism’s adaptive capacity can be harmful [55]. SANA^®^ (Risskov, Denmark) utilizes a personalized and algorithm-based treatment protocol for intermittent normobaric hypoxia–hyperoxia conditioning (IHHC) to obtain the most accurate hypoxic stimuli for each individual.

The aim of the present study was to evaluate the effects of the SANA^®^ Therapy algorithm and the subsequent individually tailored IHHC treatment on quality of life and pain in a consecutive cohort of patients suffering from PCC.

## 2. Materials and Methods

### 2.1. Design

SANA^®^ Therapy was designed by a Danish medical company specializing in algorithm-based individualized intermittent hypoxic–hyperoxic conditioning (IHHC) for various conditions, administered at five clinics in Denmark (SANA Medical Systems ApS, Risskov, Denmark).

All treatment sessions were preceded by a structured medical interview conducted by one of SANA’s physicians. Prior to the interview, patients received online questionnaires to fill out at home, consisting of questions regarding age, gender, height, weight, occupation, work absence (yes/no), symptoms, duration of symptoms, prior treatment modalities, and pain experienced during rest and activity in the past week on a numeric rating scale (NRS) from 0 (“no pain”) to 10 (“worst imaginable pain”), as well as the health-related Short Form 36 quality of life questionnaire (SF-36). Both the NRS and the SF-36 are internationally validated and standardized questionnaires to evaluate pain and health-related quality of life, respectively. The diagnosis was obtained by the physician based exclusively on the patient’s medical history and their self-reported perception of a causal relationship between symptom onset and a prior SARS-CoV-2 infection confirmed by either a molecular PCR test or a rapid antigen test. Physicians considered variations in symptom presentation, including the timing of onset (e.g., symptoms appearing immediately after infection versus weeks later), severity (mild fatigue versus debilitating exhaustion), and progression (persistent versus relapsing–remitting symptoms). These variations were assessed in conjunction with patients’ reports of functional impairment and symptom clusters commonly associated with post-COVID condition (PCC), such as cognitive dysfunction, shortness of breath, and autonomic dysfunction. An algorithm-based individualized approach was used to determine adjustable parameters (duration of intervals, number of intervals, total hypoxic exposure time, and O_2_ saturation target) for the first treatment session based on the questionnaire data.

Follow-up questionnaires were automatically sent at 6 weeks and 6 months after the first treatment session. Only one questionnaire was sent at each time point, and no reminders were provided.

Contraindications for treatment initiation were pregnancy or the presence of malignant disease. Patients younger than 18 years of age were allowed to receive treatments after individual evaluation and consent from their parents and/or legal guardian.

Patients sought treatment voluntarily, without official referrals from general practitioners, COVID-19 clinics, or hospital departments. Treatment was either self-funded per session or covered by medical insurance for rehabilitation. Participation was strictly voluntary, allowing patients to reschedule appointments or discontinue the treatment plan at any time. They were also permitted to continue or start any other treatments or pursue additional or alternative therapies simultaneously.

### 2.2. Treatment Algorithm

SANA^®^ Therapy consists of individualized treatment sessions of IHHC. A machine learning-based algorithm (version 1.0) was developed from data on 756 patients who received IHHC in doses prescribed by three physicians specializing in IHHC (co-authors RS, BE, and CF). This algorithm was trained using a random forest machine learning approach, incorporating patient demographics, diagnoses, symptoms, and the NRS and SF-36 scores at baseline. Based on these features, the algorithm determines the optimal treatment intensity and subsequently prescribes oxygen saturation levels and total hypoxia exposure, quantified as the integral of time spent below 90% saturation.

### 2.3. Treatment

SANA^®^ Therapy employs medical-grade hypoxicators (HypoxBreath^®^ (INVATIO, Hannover, Germany) and CellOxy (TUR, Rostock, Germany)), which separate oxygen and nitrogen from the atmospheric air at standard pressure to generate a controlled gas mixture with a defined fraction of inspiratory oxygen (FiO_2_). The devices use membrane filtration to modulate nitrogen–oxygen separation, allowing for precise FiO_2_ regulation during treatment cycles.

Patients, seated in a reclining chair, received the modified gas mixture through a face mask with headgear. Treatment consisted of IHHC sessions with hypoxic intervals of 3–8 min (FiO_2_ 9–13%) and hyperoxic intervals of 1–3 min (FiO_2_ 34–36%), targeting SpO_2_ levels of 77–84%, continuously monitored by a pulse oximeter. The breathing circuit is a closed-loop system with integrated valves that regulate the gas flow to ensure consistency in oxygen delivery.

The session duration ranged from 18 to 42 min, with the total number of sessions determined by symptom progression. The treatment system was validated through technical assessments to ensure reproducibility and accuracy in oxygen regulation across all sessions.

### 2.4. Statistical Analysis

Patient data were extracted from the SANA^®^ consecutive database consisting of all patients initiating treatment at SANA^®^ Clinics in Denmark from 1 January 2020 to 31 December 2023, allowing for a 6-month follow-up period. Exclusion criteria for the statistical analysis were symptom durations of less than three months, the absence of fatigue (defined as an SF-36 vitality domain > 50 at baseline), or the receipt of only the initial baseline treatment. Each follow-up cohort was analyzed individually, with data paired from baseline to follow-up for each patient.

Statistical analyses were performed using Prism 10 (v10.2.3, GraphPad Software LLC). Data normality was assessed via QQ plots of residuals. A one-way ANOVA with Sidak’s correction for multiple comparisons was applied for the demographic group and SF-36 evaluations. Despite the previous debate on combining the four mental and four physical domains in a total score [56], we included a surrogate quality of life score, calculated as the sum of the eight domains at each time point. For pain evaluation, only patients reporting NRS ≥ 1 at rest or during activity at baseline were included, analyzed using a one-way ANOVA with Sidak’s multiple comparisons in each cohort. For intergroup comparisons, Chi-squared tests were used to assess gender distribution differences. A significance level of *p* < 0.05 was applied. Results are reported as mean absolute values with ±95% confidence intervals [±95% CI]. Baseline-relative changes are stated in parentheses.

### 2.5. Ethics

The study was approved by the local ethics committee under the Danish National Committee of Research Ethics (# 1-10-72-274-21). The study was conducted in accordance with the Declaration of Helsinki, and all participant data were handled in compliance with the EU General Data Protection Regulation (GDPR).

## 3. Results

### 3.1. Patients and Treatments

**Figure 1:**
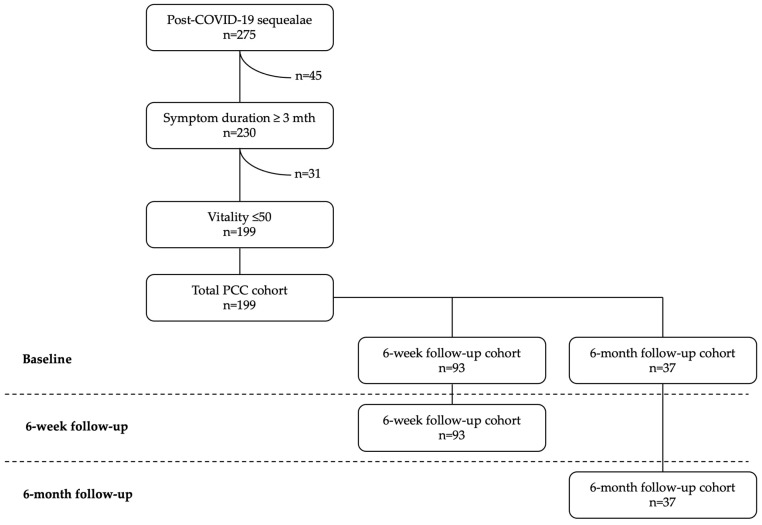
Inclusion flow-chart and study design. All patients completed baseline questionnaires before treatment initiation. Patients who submitted the 6-week and/or 6-month follow-up questionnaires were included in the 6-week (6 w) and 6-month (6 m) cohorts, respectively. The diagnosis of PCC was based on the patients’ self-reported symptom onset following SARS-CoV-2 infection with a symptom duration exceeding 3 months. As fatigue is the most common symptom of PCC, its absence was used as an exclusion criterion in the statistical analysis. Antibody and/or antigen tests were not used to confirm or validate of the diagnosis prior to treatment initiation.

A total of 275 patients with PCC were identified in our consecutive database. Forty-five patients were excluded due to a symptom duration of less than three months, and 31 patients were excluded due to lack of moderate or severe fatigue. Ten patients only received the initial baseline treatment, but these patients all had a symptom duration of less than three months and were excluded through this criterion. Consequently, 199 patients were included in the study (Figure 1), constituting the total PCC cohort.

Ninety-three patients filled out the questionnaire sent out at 6 weeks after the first treatment session (reporting rate of 46.7%), while only 37 of these 93 patients reported their health status at the 6-month follow-up (reporting rate of 18.6%). To enable a paired analysis, follow-up data were matched with baseline data, establishing a 6-week (6 w) and a 6-month (6 m) cohort for subsequent analysis (Figure 1). The group demographics are presented in Table 1.

The median number of treatment sessions was six (range 2–21), with 65% of patients receiving three to seven treatments (Supplementary Figure S1). The most prevalent prior treatment attempt was non-prescription analgesics, while nearly half of the patients had previously undergone physiotherapy for their symptoms (Supplementary Table S1).

### 3.2. Quality of Life Outcome

Quality of life was evaluated using the SF-36 health survey, which consists of 36 questions assessing eight health domains, each scored from 0 to 100, with higher scores reflecting better health. An additional measure, the self-reported health transition over the past year, was collected but not included in the total score. A health transition score below 50 indicates a perceived decline in health compared to one year ago, a score of 50 signifies no change, and a score above 50 reflects an improvement in health over the past year [57].

At baseline, the overall quality of life scores in the PCC patient cohort were low (Table 2). The mean total score across the total cohort was 380, with the 6-week and 6-month follow-up cohorts having baseline scores of 357 and 389, respectively. For comparison, a Danish study from 1998 reported a mean total score in the population of 645 [58]. In all three cohorts, the baseline scores were particularly low in the role physical domain (ranging between 9.95 and 14.2) and vitality domain (ranging between 17.1 and 22.4). No significant differences were observed between the SF-36 domain scores among the three groups at baseline.

**Figure 2:**
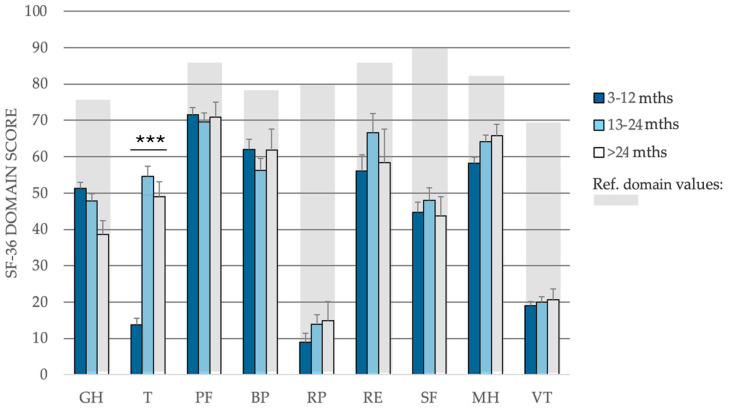
SF-36 domain scores at baseline in the total PCC cohort depending on the duration of symptoms. Reference values for the Danish population are shown in light grey boxes [58]. Eight SF-36 health domains were analyzed: general health (GH), health transition (T), physical functioning (PF), bodily pain (BP), role physical (RP), role emotional (RE), social functioning (SF), mental health (MH), and vitality (VT). Data are presented as mean values, with error bars representing the standard error of the mean (SEM). Health transition scores (T) of patients with a symptom duration of 3–12 months differed significantly compared to the scores of patients with a symptom duration of 13–24 months and >24 months. *** = *p* < 0.001.

To further characterize the total PCC cohort at baseline, the 199 patients were categorized into three groups based on the duration of their symptoms: patients with symptoms that lasted 3–12 months (n = 104; 52.3%), 13–24 months (n = 71; 35.7%), and more than 24 months (n = 24; 12.1%). The SF-36 health transition score was low in the group with a symptom duration of 3–12 months, with a mean of 13.7 [95% CI, 10.1–17.3], reflecting a substantial decline in the self-assessed health status compared to their pre-infection state (Figure 2). Interestingly, the two patient groups with symptoms lasting more than 12 months had health transition scores closer to 50 (54.6 [95% CI, 49.1–60.1] and 49.0 [95% CI, 40.4–57.5], respectively), which suggests no perceived change in health status over the past year and reflects the lack of symptom resolution in PCC patients. However, the duration of symptoms before the initiation of SANA^®^ Therapy did not affect the treatment outcomes.

**Figure 3:**
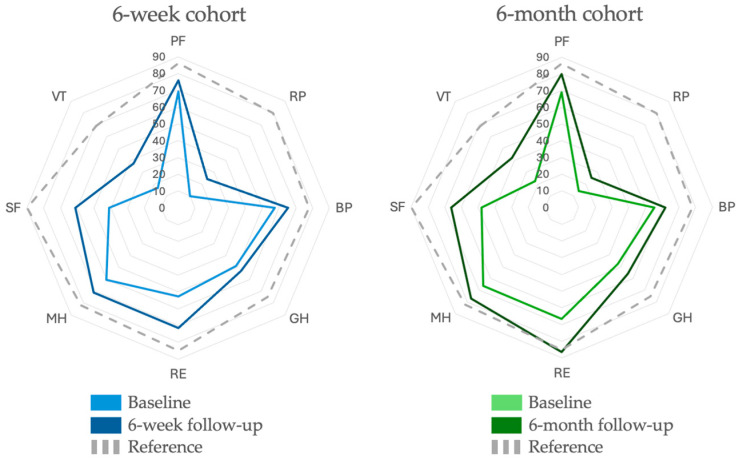
SF-36 domain scores in the 6-week (**left**) and 6-month (**right**) follow-up cohorts. Baseline values are illustrated in light blue and green, and follow-up values (either 6 weeks or 6 months after treatment initiation) are illustrated in dark blue and green. Dashed lines represent reference values for the general Danish population [58]. The eight SF-36 health domains are included: physical functioning (PF), role physical (RP), bodily pain (BP), general health (GH), vitality (VT), social functioning (SF), role emotional (RE), and mental health (MH).

Significant improvements were observed in all SF-36 domains from baseline to the 6-week follow-up, except for general health, which did not reach statistical significance at the 5% level (Table 2). At the 6-month follow-up, the improvements remained significant in all domains except role physical, bodily pain, and role emotional. Six weeks and six months after treatment initiation, the total SF-36 score was increased by 102 points (*p* < 0.001) [95% CI, 78.4–127] and 106 points (*p* < 0.001) [95% CI, 57.0–154], respectively. Changes in quality of life, assessed by the eight SF-36 parameters, are also illustrated in Figure 3.

The health transition scores improved significantly from below 50 at baseline to above 50 at the 6-week and 6-month follow-ups (*p* < 0.001), with increases of 22.6 points [95% CI, 14.0–31.2] and 31.1 points [95% CI, 14.4–47.8], respectively.

### 3.3. Pain

Pain was analyzed in patients with a baseline NRS score of ≥1 at rest and/or during activity. At baseline, 80.4% of the patients reported pain at rest, with a mean intensity of 4.5 [95% CI, 4.16–4.83]. During activity, 83.9% of patients reported pain, with a mean intensity of 5.3 [95% CI, 5.10–5.79]. No gender differences were observed. Headache was identified as the origin of pain in 53.8% of patients at rest and 53.9% during activity. There was no difference in pain intensity between patients with headache and those with non-headache pain, either at rest or during activity.

**Figure 4:**
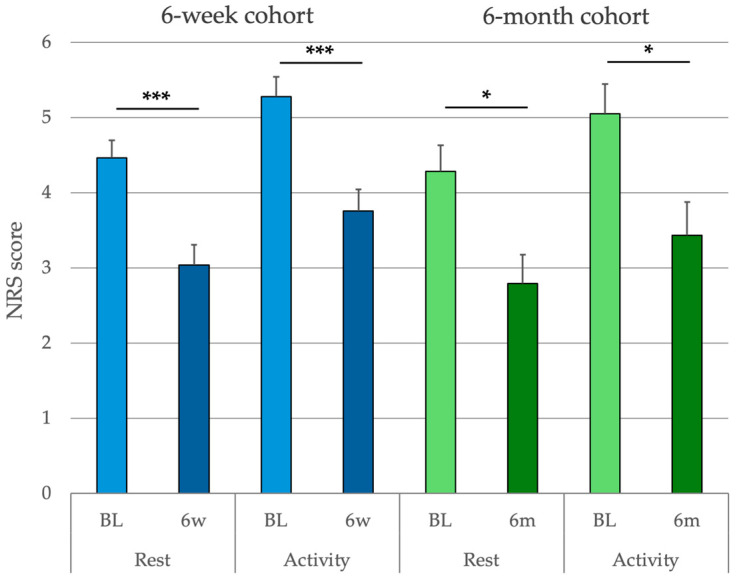
NRS pain scores at rest and during activity. Mean reduction in NRS pain scores in the 6-week (6 w) and 6-month (6 m) follow-up cohorts. Pain during rest and activity is shown. Error bars represent standard deviations. Pain reduction was significant during activity and at rest at both follow-up time points. The reduction exceeded the clinically relevant threshold of 0.9. Significance levels are denoted as * = *p* < 0.05 and *** = *p* < 0.001.

From baseline to the 6-week follow-up, the mean reduction in pain at rest was 1.43 NRS points [95% CI, 0.90 to 1.95] (mean pain reduction of 32.0%; *p* < 0.0001), and that during activity was 1.52 [95% CI, 0.99 to 2.06] (mean reduction of 28.9%; *p* < 0.0001). The mean reduction in pain at rest from baseline to 6 months was 1.49 [95% CI, −0.46 to 2.51] (mean reduction of 34.5%; *p* < 0.01) and that during activity was 1.62 [95% CI, −0.41 to 2.83] (mean reduction of 32.0%; *p* < 0.05). No significant differences were found between the 6-week and 6-month follow-up cohorts (Figure 4).

## 4. Discussion

This study demonstrates the potential of algorithm-based, personalized intermittent hypoxia–hyperoxia conditioning (IHHC) to enhance quality of life and relieve pain in patients suffering from COVID-19 sequelae. In the present cohort of 199 PCC patients, the SF-36 domains were notably low at baseline, consistent with previous studies on PCC patients [59,60,61]. Six weeks after treatment initiation with IHHC, significant improvements were observed in pain and all SF-36 health domains except for general health, and the improvements persisted at the 6-month follow-up. The SF-36 total score (ranging from 0 to 800) increased by 102 points (*p* < 0.001) and 106 points (*p* < 0.001) at the 6-week and 6-month follow-up time points. This reflects the large increase in self-perceived health-related quality of life of 27–29% at the two time points. Remarkably, the health transition scores improved considerably, reaching 69.6 at the six-month follow-up, indicating a shift from self-perceived health decline to health improvement over the past year.

As a multisystemic disease, more than 200 symptoms have been identified in PCC [61]. Among these, post-exertional malaise (PEM) is the most prevalent, reported in 89.1% of a cohort of 3762 patients [62]. Likewise, the RECOVER study led by the NIH found that 87% of patients suffered from PEM, making it the most common symptom of PCC [10]. A recent study found that patients with fatigue and meeting the criteria for chronic fatigue syndrome/myalgic encephalomyelitis reported more persistent and severe symptoms over a 20-month follow-up period, compared to those without severe fatigue [63]. While PEM affects all domains of the SF-36 evaluation, the vitality and role physical domains are often used to assess these patients’ energy levels, fatigue, and perceived physical functioning [64]. The current patient cohort likewise reported low scores in these domains (19.5 and 11.4, respectively), considerably below the scores of 69 and 80 in the Danish population [58]. Following the IHHC regimen, twofold improvements were observed in both domains. As PEM may serve as a diagnostic marker for symptom severity and stagnation over 20 months [63], it is important to emphasize that our patient group had undergone various other treatments with inadequate effects and reported health declines or stagnation over the last year before trying SANA^®^ Therapy.

Limited studies are available that evaluate the effects of IHC or IHHC in the treatment of the sequelae of COVID-19 infections. A randomized, single-blinded study of 95 PCC patients found that the 6-min walking distance, lung function, and fatigue improved in the IHC group compared to controls [65]. Similarly, another controlled interventional study from September 2024 investigated 145 PCC patients undergoing either a comprehensive standardized rehabilitation program combined with IHHC or the rehabilitation program alone [66]. Significant improvements in exercise capacity, dyspnea, fatigue, quality of life, blood pressure, and heart rate were observed in the IHHC group, with 72% of patients reporting considerably improved symptoms, compared to only 15.2% in the control group undergoing standard treatment [66]. As in both trials, no severe side effects were reported in the current study; the most common adverse events were mild headaches and tiredness lasting 12–24 h, typically resolving after the initial treatments as the patients developed a tolerance to the hypoxic stimulus. IHHC therapy thus emerges as a safe, minimally invasive option for PCC rehabilitation.

A limitation of this study was the low 6-month follow-up rate, which could have been increased by sending reminders to patients. The low response rate may have introduced reporting bias, although it can be questioned whether the responders were the ones who experienced symptom alleviation or a lack of effect of treatment; therefore, it remains unknown in which direction this may have influenced the results of the current study. No differences in baseline characteristics were observed, limiting the risk of bias. A placebo group was not included in the present study, and the placebo effect may have contributed to the observed outcomes. As all patients were either self-paying or referred by their health insurance company, this may have contributed to placebo effects due to expectations, self-justification, and general biopsychosocial mechanisms. Additionally, self-payment might have led some patients to discontinue treatment before achieving the maximal effect due to financial constraints. Therefore, self-payment could have influenced the results in opposing directions, highlighting the need for further studies that eliminate economic factors as a confounding variable. It should also be noted that patients reported health declines or stagnation in the year prior to treatment initiation, minimizing the likelihood that the observed effects of IHHC were merely due to the evolution of time. Patients were not prohibited from continuing or initiating other treatments or lifestyle changes, which represented a limitation in the study, leaving the potential effects of combined therapies unexplored. However, this also enhances the study’s external validity by reflecting real-world conditions. Further research is needed to determine whether longer IHHC regimens or multimodal approaches can fully restore quality of life in PCC patients.

The primary strength of this study lies in its large consecutive cohort with diverse demographics, strengthening the external validity of the results. Despite the relatively low 6-month follow-up rate, the findings suggest a sustained long-term effect of IHHC in treating the sequelae of COVID-19 infections. Additionally, this study is the first to evaluate the effects of personalized IHHC in treating PCC patients, utilizing a state-of-the-art AI-based algorithm to predict the most effective treatment regimen for each individual. While questionnaire-based results are accompanied by certain limitations, this approach is also a relevant and pragmatic way to evaluate real-world outcomes by placing the patient at the center, rather than relying solely on objective data such as serology.

In conclusion, our results demonstrate that SANA^®^ Therapy—an AI-based approach to individualized IHHC—significantly improves quality of life and reduces pain for up to six months in PCC patients who have a mean symptom duration of 14.1 months prior to treatment. Thereby, SANA^®^ Therapy may represent a cost-effective treatment for individuals suffering from long-term sequelae of COVID-19 infection by enhancing their quality of life, alleviating fatigue, and reducing persistent pain. This innovative approach holds promise as a valuable therapeutic option to address the prolonged health impacts of COVID-19.

## 5. Patents

No patents are disclosed in this manuscript. However, the SANA^®^ Therapy algorithms are proprietary, confidential, and remain the exclusive intellectual property of SANA Medical Systems ApS, Risskov, Denmark.

## Figures and Tables

**Table 1:** Demographics and baseline characteristics in the three cohorts. Data are presented as the total PPC cohort, 6-week follow-up cohort (6 w), and 6-month follow-up cohort (6 m). Values are reported as means with standard deviations in parentheses. “Sick leave” represents baseline values of patients included in the respective follow-up cohorts. Patients in the 6-month follow-up cohort were significantly older and had significantly longer symptom durations compared to baseline.

	Total PCC	6 w	6 m
Number of patients	199	93	37
Age	46.8 (±13.1)	47.9 (±13.1)	52.4 (±11.0) *
Gender (M:F)	33:67	38:62	22:78
Height (cm)	173.5 (±9.3)	174.2 (±9.7)	173.0 (±10.2)
Weight (kg)	76.7 (±16.5)	78.3 (±17.0)	75.5 (±16.5)
Duration of symptoms (months)	14.1 (±8.4)	11.7 (±10.2)	18.2 (±13.7) *
Sick leave (%) at baseline	60.3	68.8	67.6

Asterix (*) indicates a *p*-value < 0.05.

**Table 2:** Effect of SANA^®^ Therapy on SF-36 domains and changes relative to baseline. Changes in domain values from baseline to follow-up (Δ) were calculated for each domain at each time point and reported as the mean ± 95% CI.

SF-36 Domain	Total PCC	Ref.	6-Week Cohort				6-Month Cohort			
	BL		BL	6 w	Δ	±95% CI	BL	6 m	Δ	±95% CI
General health	48.5	75	48.8	53.0	4.19	−0.14; 8.52	47.4	55.9	8.51 **	1.51; 15.1
Physical functioning	73.3	86	69.3	75.9	6.65 ***	2.53; 10.8	68.9	80.0	11.1 ***	2.66; 19.5
Role physical	11.4	80	9.95	24.2	14.3 ***	4.85; 23.7	14.2	25.0	11.8	−8.63; 30.3
Bodily pain	60.0	78	57.6	65.2	7.69 **	2.19; 13.2	55.3	62.0	6.69	−4.17; 17.5
Mental health	61.2	82	60.5	71.1	10.6 ***	6.00; 15.2	66.3	76.9	10.6 **	2.90; 18.3
Role emotional	60.1	85	52.7	71.3	18.6 **	3.61; 33.7	66.7	86.5	19.8	−1.83; 41.5
Social functioning	45.7	90	41.1	61.1	20.1 ***	13.5; 26.6	47.9	66.3	18.4 **	5.76; 31.0
Vitality	19.5	69	17.1	37.6	20.5 ***	14.5; 26.5	22.4	42.2	19.7 ***	8.48; 31.0
Health transition	32.5	N/A	29.3	51.9	22.6 ***	14.0; 31.2	38.5	69.6	31.1 ***	14.4; 47.8
Total score	380	645	357	459	102 ***	78.4; 127	389	495	106 ***	57.0; 154

Significance is denoted as ** *p* < 0.01 and *** *p* < 0.001. Reference values (Ref.) for the general Danish population were obtained from [58]. BL = baseline; 6 w = 6-week follow-up after treatment initiation; 6 m = 6-month follow-up after treatment initiation.

## Data Availability

The data is part of the SANA^®^ consecutive database and is not publicly available.

## Supplementary Materials

The following supporting information can be downloaded at: https://www.mdpi.com/article/10.3390/jcm14051590/s1, Figure S1: Number of total treatment sessions per patient depicted as prevalence in percentages; Table S1: Prevalence of prior treatment modalities at baseline.

## Abbreviations

The following abbreviations are used in this manuscript:

AI	Artificial Intelligence
ANOVA	Analysis of Variance
CI	Confidence Interval
DOAJ	Directory of Open Access Journals
FiO2	Fraction of Inspired Oxygen
GDPR	General Data Protection Regulation
IHC	Intermittent Hypoxia Conditioning
IHHC	Intermittent Hypoxia–Hyperoxia Conditioning
MDPI	Multidisciplinary Digital Publishing Institute
NIH	National Institutes of Health
NRS	Numeric Rating Scale
PACS	Post-Acute Sequelae of COVID-19
PCC	Post-COVID-19 Condition
PEM	Post-Exertional Malaise
PCR	Polymerase Chain Reaction
SARS-CoV-2	Severe Acute Respiratory Syndrome Coronavirus Type 2
SF-36	Short Form-36 Health Survey
WHO	World Health Organization
