# Supplementary material for: Individualized Algorithm-Based Intermittent Hypoxia Improves Quality of Life in Patients Suffering from Long-Term Sequelae After COVID-19 Infection
Source: J Clin Med. 2025 Feb 26;14(5):1590. doi: 10.3390/jcm14051590 (PMC11900126; doi:10.3390/jcm14051590)
Supplement: Supplementary file 1 [file jcm-14-01590-s001.zip › jcm-3468853-supplementary.pdf]

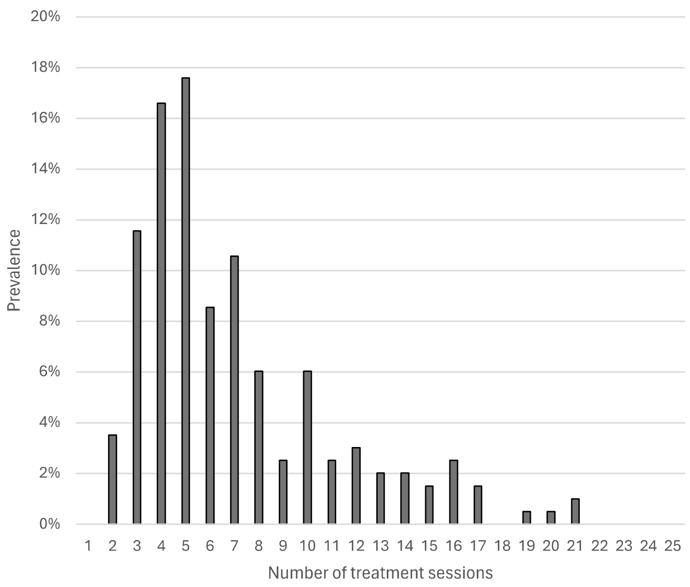

Figure S1 Number of total treatments sessions per patient depicted as prevalence in percentage. Sixty-five percent of the patients received 3-7 sessions while 34% received 4-5 sessions.

Table S1. Prevalence of prior treatment modalities at baseline in the total PCC cohort (n=199).

Type of treatment	No. of patients	Prevalence
Analgesics: non-prescription	108	54.3%
Physiotherapy	88	44.2%
Acupuncture	62	31.2%
Analgesics: prescription	44	22.1%
Osteopathy	35	17.6%
Chiropractor	30	15.1%
Ergotherapy	20	10.1%
Craniosacral therapy	19	9.5%
Massage	16	8.0%
Phycologist	11	5.5%
